# Mibefradil alters intracellular calcium concentration by activation of phospholipase C and IP_3_ receptor function

**DOI:** 10.1186/s43556-021-00037-0

**Published:** 2021-04-30

**Authors:** Guilherme H. Souza Bomfim, Erna Mitaishvili, Talita Ferreira Aguiar, Rodrigo S. Lacruz

**Affiliations:** 1 Department of Molecular Pathobiology, New York University College of Dentistry, New York, NY 10010 USA; 2 Department of Urology, New York University School of Medicine, New York, NY 10010 USA

**Keywords:** Ca^2+^ signaling, Mibefradil, PLC pathway, Ca_v_, LS8 cells, ALC cells, HEK293 cells

## Abstract

Mibefradil is a tetralol derivative originally developed as an antagonist of T-type voltage-gated calcium (Ca^2+^) channels to treat hypertension when used at nanomolar dosage. More recently, its therapeutic application in hypertension has declined and has been instead repurposed as a treatment of cancer cell proliferation and solid tumor growth. Beyond its function as a Ca_v_ blocker, the micromolar concentration of mibefradil can stimulate a rise in [Ca^2+^]_cyt_ although the mechanism is poorly known. The chanzyme TRPM7 (transient receptor potential melastanin 7), the release of intracellular Ca^2+^ pools, and Ca^2+^ influx by ORAI channels have been associated with the increase in [Ca^2+^]_cyt_ triggered by mibefradil. This study aims to investigate the cellular targets and pathways associated with mibefradil’s effect on [Ca^2+^]_cyt_. To address these questions, we monitored changes in [Ca^2+^]_cyt_ in the specialized mouse epithelial cells (LS8 and ALC) and the widely used HEK-293 cells by stimulating these cells with mibefradil (0.1 μM to 100 μM). We show that mibefradil elicits an increase in [Ca^2+^]_cyt_ at concentrations above 10 μM (IC_50_ around 50 μM) and a fast Ca^2+^ increase capacity at 100 μM. We found that inhibiting IP_3_ receptors, depleting the ER-Ca^2+^ stores, or blocking phospholipase C (PLC), significantly decreased the capacity of mibefradil to elevate [Ca^2+^]_cyt._ Moreover, the transient application of 100 μM mibefradil triggered Ca^2+^ influx by store-operated Ca^2+^ entry (SOCE) mediated by the ORAI channels. Our findings reveal that IP_3_R and PLC are potential new targets of mibefradil offering novel insights into the effects of this drug.

**Supplementary Information:**

The online version contains supplementary material available at 10.1186/s43556-021-00037-0.

## Introduction

Mibefradil, also known as Ro 40–5967, is an organic compound derivative of tetralol that has been used as a Ca^2+^ channel antagonist (CCA). Patch-clamp studies revealed that mibefradil has an IC_50_ around 0.3–2.7 μM for Ca^2+^, Na^+,^ and K^+^ voltage-dependent currents in HEK-293 cells [1–3]. Mibefradil, a T-type voltage-gated calcium (Ca^2+^) channels (Ca_v_ 3.1–3.3) inhibitor, was launched by Roche as Posicor® for the treatment of hypertension and stable angina at doses containing 50–100 mg once a day [1, 4]. The maximum plasma circulating levels of mibefradil were around 1 μg/mL after 1–2 h of the single 100 mg dose [4, 5]. Although mibefradil has a potent effect on T-type CCA, being 10- to 30-fold higher for T-type than L-type [4], adverse clinical outcomes resulted in its withdrawal from the market in 1998 because of drug-drug interactions, side-effects, and inhibition of cytochrome P450 3A4 [6].

Recently, mibefradil has seen a renewed pharmacological interest being an FDA “orphan drug” approved for its efficacy in cancer management [6–8]. Mibefradil has been used to treat ovarian and pancreatic cancer, glioblastoma and has an anti-proliferative effect on several cancer cell lines [8–13]. In melanoma cells, the anti-proliferative and anti-tumoral properties of mibefradil were dependent on caspase activation, the transfer of Ca^2+^ into the endoplasmic reticulum (ER) [14, 15], and the inhibition of basal macroautophagy which is constitutively active in melanoma cells [16]. In addition, retinoblastoma and glioma cells exposed to micromolar concentrations (≥10 μM) of mibefradil revealed an increase in the number of cells in the G1 phase and a decrease in the number of cells in the S-phase in a dose-dependent manner [12, 17].

From the pharmacological viewpoint, in vitro studies show that the use of mibefradil at micromolar concentrations (10–100 μM) elicits an increase in [Ca^2+^]_cyt_ although the molecular mechanism involved is unclear [18–20]. Possibilities include a slow recovery from the opening of the Ca_v_ block [21] or the activation of the Mg^2+^ channel TRPM7 (transient receptor potential melastanin 7) that is permeable to Ca^2+^ [20]. In HEK-293 cells overexpressing TRPM7 channels, mibefradil (100 μM) increased [Ca^2+^]_cyt_ that was abolished by perfusing with the TRPM7 antagonist NS8593 [20]. Another potential contributor associated with the elevation in [Ca^2+^]_cyt_ elicited by mibefradil is the release of Ca^2+^ from intracellular stores [18] mediated by the activation of inositol 1,4,5-triphosphate (IP_3_) receptors (IP_3_R) [19].

Physiologically, the release of Ca^2+^ pools from the endoplasmic reticulum (ER) is generated through a family of *G* protein-coupled (GPCRs) and/or enzyme-linked receptors at the cell membrane in response to an agonist activating primary (β-γ) and/or secondary (δ) phospholipases C (PLC) isozymes, a group of Ca^2+^-dependent phosphodiesterases hydrolyzing phosphatidyl-inositol bisphosphate (PIP_2_) into 1,2-diacylglycerol (DAG) and (IP_3_) [22]. DAG and/or cell depolarization elicits Ca^2+^ influx through of the functional L-type Ca_v_ 1.2 and T-type Ca_v_ 3.1–3.3 [22, 23]. The binding of IP_3_ to its receptors in the ER membrane releases Ca^2+^ pools via the IP_3_R channels subtype 1, 2, and 3 [23]. This step stimulates Ca^2+^ uptake from extracellular sources via the store-operated Ca^2+^ entry (SOCE) pathway that is mediated by the Ca^2+^release-activated Ca^2+^ (CRAC) channel components STIM1/2 and ORAI1–3, replenishing the ER Ca^2+^ stores and activating Ca^2+^-dependent pathways [24]. Electrophysiological studies reported that mibefradil blocked CRAC currents in a concentration-dependent manner (10–100 μM) in HEK-293 cells overexpressing STIM-ORAI1–3 [25]. This may suggest an interaction with the ORAI channels when acutely perfused. Pretreatment with mibefradil (100 μM) for 24 h abolished ER-Ca^2+^ release and Ca^2+^ influx [25]. Additionally, Johnson et al. demonstrated that 20 μM mibefradil increases [Ca^2+^]_cyt_ via STIM1 and ORAI-1 [26]. Thus, addressing the effects of mibefradil in [Ca^2+^]_cyt_ and its impact on the CRAC channels is important to better understand the broader biological effects of this drug.

Here, we have investigated the cellular targets and pathways associated with the transient application of mibefradil and its effect on [Ca^2+^]_cyt_ in vitro. Using the murine epithelial cells LS8 and ALC, and the human HEK-293 cells that are a generalized cell model widely used in signal transduction research [27], we show that mibefradil (0.1 μM to 100 μM) elicited a significant increase in [Ca^2+^]_cyt_ in all cell lines. We found that this effect was reduced or abolished by inhibiting IP_3_R or PLC, or by depleting the ER-Ca^2+^ stores. Mibefradil also triggered SOCE, likely as a result of its role in activating the PLC pathway. Taken together, our findings suggest that the application of mibefradil at micromolar (≥10 μM) concentrations elevates [Ca^2+^]_cyt_, highlighting its effects on the PLC pathway and the ER-Ca^2+^ released via the IP_3_R as potential targets thus providing novel insights into the effects of mibefradil.

## Results

### Mibefradil caused a concentration-dependent increase in [Ca^2+^]_cyt_

**Fig. 1:**
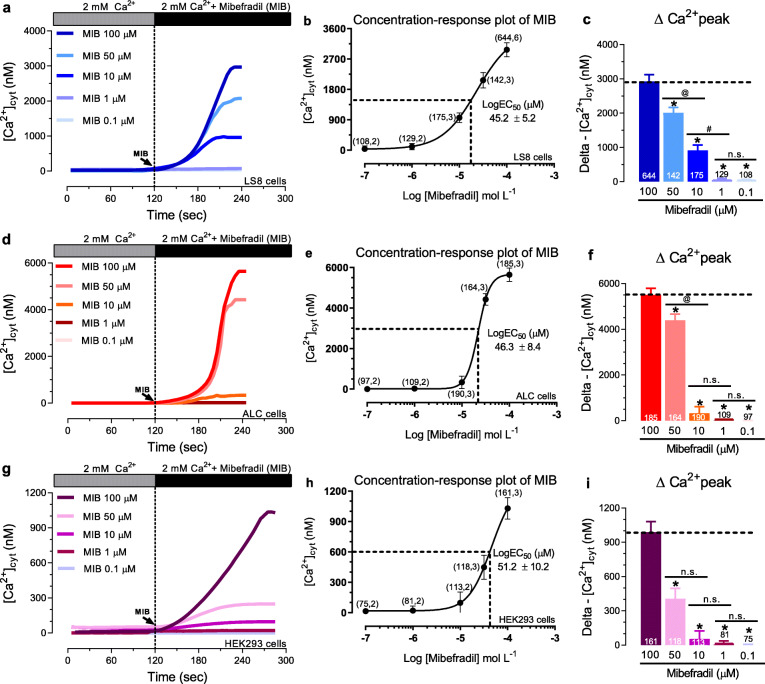
Effects of mibefradil on [Ca^2+^]_cyt_ transients in LS8, ALC, and HEK-293 cells. **a** [Ca^2+^]_cyt_ transients in LS8 cells stimulated with mibefradil (0.1 μM to 100 μM) in the presence of 2 mM extracellular Ca^2+^. **b** LogEC_50_ (apparent affinity) plot. **c** Quantification of ∆Ca^2+^ peak. **d-f** Same as in a-c but measured in ALC cells. **g-i** Same as a-c but measured in HEK-293 cells. In all cells, traces show basal (0–120 s) Ca^2+^ levels and the maximum capacity of Ca^2+^ mobilization (240–300 s) at 100 μM. Data represent the mean ± SEM of ≥75 cells from 3 independent experiments. Data were analyzed by one-way ANOVA followed by Tukey’s multiple comparison post-hoc test. *P < 0.05 vs. MIB-100 μM group; ^**@**^*P* < 0.05 vs. MIB-50 μM group; ^**#**^*P* < 0.05 vs. MIB-10 μM group; n.s., non-significant

To determine the maximum capacity of Ca^2+^ mobilization by mibefradil, we constructed concentration-effect curves (0.1–100 μM) in murine LS8 and ALC and in human HEK-293 cell lines (Fig. 1). In an extracellular 2 mM Ca^2+^ solution, the perfusion of 0.1–1 μM mibefradil, commonly used to block Ca_v_ 1.2–3.3 [4], had no effect in [Ca^2+^]_cyt_ in any of the cell lines. By contrast, at concentrations above 10 μM, mibefradil raised [Ca^2+^]_cyt_ from nanomolar to micromolar (~ 1–5 μM) levels, with a maximum Ca^2+^ increase at 100 μM (Fig. 1). No background autofluorescence was detected in the Ringer’s solution with or without mibefradil (50 μM, 100 μM) (Fig. S1). Although LS8, ALC, and HEK-293 cells showed differences in the maximum capacity of Ca^2+^ transients, they all revealed similar values of LogEC_50_ (~ 45–51 μM) (Fig. 1b, e, h). The efficiency of mibefradil to elevate [Ca^2+^]_cyt_ is demonstrated by a loss of the signal when mibefradil is removed from the solutions (Fig. S2). To ascertain if TRPM7 channels are a potential target of mibefradil, we used the selective TRPM7 agonist naltriben (100 μM) [28]. Under resting extracellular Ca^2+^ levels (2 mM Ca^2+^), naltriben stimulation did not elicit an increase in [Ca^2+^]_cyt_ in LS8 and ALC cells, although it caused a small elevation (∼30 nM) in HEK-293 cells (Fig. S3). Taken together, these data suggest that TRPM7 channels are not the main target of mibefradil although these channels may have a small contribution. Our results, together with previous reports [18–20, 29], showed that mibefradil stimulates an increase in [Ca^2+^]_cyt_ when used above 10 μM, with a maximum [Ca^2+^]_cyt_ increase at 100 μM. Thus, we used 100 μM mibefradil to study its effects on Ca^2+^ transients and possible molecular targets.

### IP_3_ levels and expression and inhibition of IP_3_ receptors

**Fig. 2:**
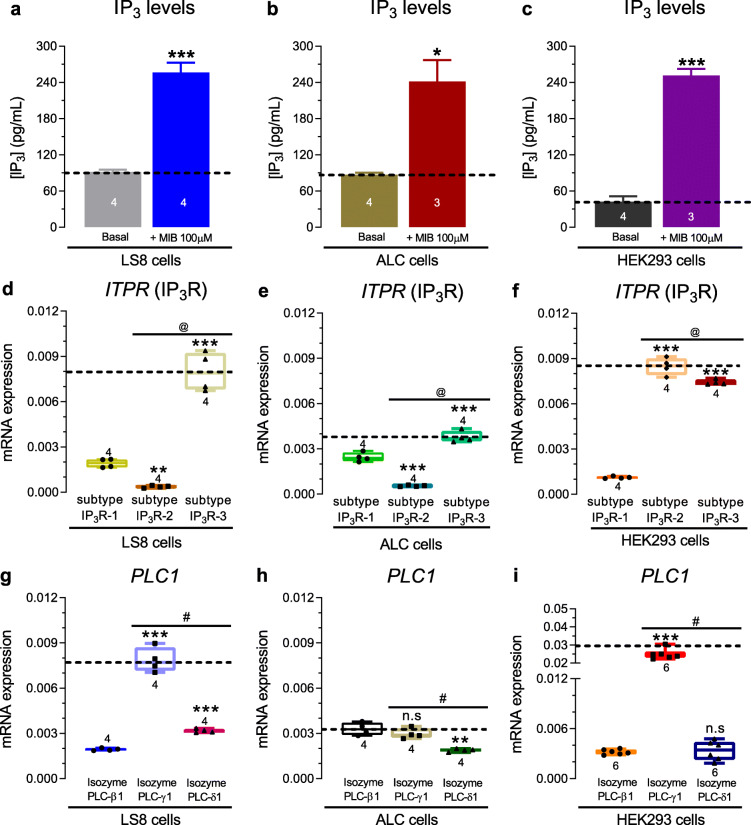
IP_3_ content and expression of IP_3_ receptor subtypes. **a** Quantification of IP_3_ content in LS8 cells. **b** Quantification of IP_3_ content in ALC cells. **c** Quantification of IP_3_ content in HEK-293 cells. In all cells, quantification was performed before and after incubation with mibefradil (100 μM, 3–4 min). **d-f** Relative mRNA expression of the *ITPR* gene encoding the IP_3_R subtypes 1–3 quantitated by qPCR in each cell type. Data represent the mean ± SEM of 3–4 independent experiments. Data were analyzed by one-way ANOVA followed by Tukey’s multiple comparison post-hoc test. *P < 0.05; **P < 0.01 or ***P < 0.001 vs. IP_3_R-1 group; ^@^*P* < 0.05 vs. IP_3_R-2 group; n.s., non-significant

**Fig. 3:**
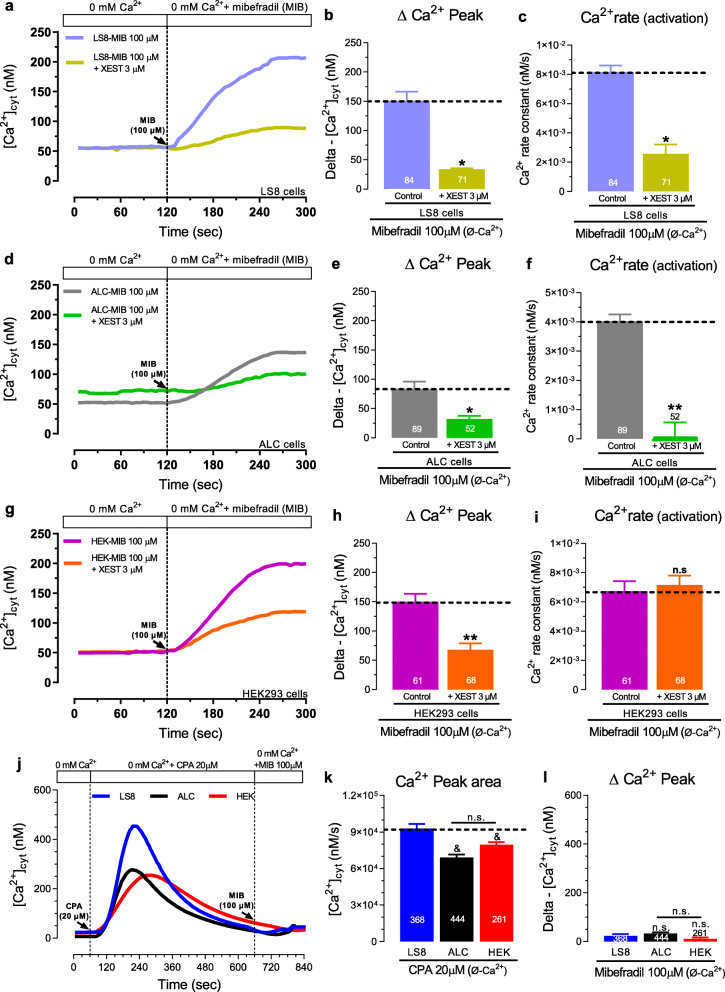
Effects of mibefradil are reduced or abolished by IP_3_ R antagonist and ER-Ca^2+^ depletion. **a** Original traces of [Ca^2+^]_cyt_ transients in Xestospongin C treated LS8 cells in Ca^2+^-free Ringer’s solution before and after mibefradil (100 μM) stimulation. **b** Quantification of the ∆ Ca^2+^ peak. **c** Quantification of the Ca^2+^ influx rate. **d-f** Same as in a-c but in ALC cells**. g-i** Same as in a-c but in HEK-293 cells**. j** Original traces of [Ca^2+^]_cyt_ transients in CPA stimulated LS8, ALC, and HEK-293 cells in Ca^2+^-free Ringer’s solution followed by the application of mibefradil (100 μM). **k** Quantification of the ∆ Ca^2+^ peak under CPA stimulation. **i** Quantification of the ∆ Ca^2+^ peak under mibefradil stimulation. Data represent the mean ± SEM of ≥52 cells from 3 independent experiments. Data were analyzed by two-tailed unpaired Student’s t-test. *P < 0.05 or **P < 0.01 vs. respective MIB-100 μM (control) group; n.s., non-significant. In ER-Ca^2+^ depletion experiments elicited by CPA (20 μM), the data were analyzed by one-way ANOVA followed by Tukey’s multiple comparison post-hoc test. ^&^*P* < 0.05 vs. LS8 group n.s., non-significant

To address the effects of mibefradil on IP_3_ signaling we first investigated if mibefradil affected the IP_3_ content in cells. We lysed cells (1.10^6^ cells/mL) of each type to measure the IP_3_ levels before and after mibefradil stimulation (100 μM for 3–4 min) (Fig. 2). Under basal conditions, the IP_3_ content was 91 ± 3.8 pg/ml in LS8, 87 ± 3.4 pg/ml in ALC, and 43 ± 8.7 pg/ml in HEK-293 cells. After mibefradil stimulation, the IP_3_ content was ∼2-fold higher in LS8 and ALC cells and ∼6-fold higher in HEK-293 cells (Fig. 2a-c). We also investigated IP_3_R expression and function. The IP_3_R subtype 3 gene (*ITPR3*) was the most highly expressed in LS8 and ALC cells but type 2 (*ITPR2*) was the most abundant mRNA in HEK-293 cells (Fig. 2d-f). To address the functional contribution of IP_3_Rs under mibefradil stimulation, we pretreated the cells with the membrane-permeable IP_3_R inhibitor Xestospongin C (3 μM, for 20 min) [30, 31] in a Ca^2+^-free Ringer’s solution. Xestospongin C drastically reduced the ∆ Ca^2+^ peak in LS8 (~ 85%), ALC (~ 70%), and HEK-293 (~ 60%) cells (Fig. 3b, e, h). This IP_3_R inhibitor also caused a slowdown in the Ca^2+^ influx rate in LS8 and ALC cells (Fig. 3c, f). These results, together with the IP_3_ content data, suggest that the rise in [Ca^2+^]_cyt_ elicited by mibefradil (100 μM) involves modulation of IP_3_ and IP_3_R.

### ER-Ca^2+^ depletion impacts the effects of mibefradil

Next, to address the role of the largest intracellular Ca^2+^ store, the ER, we passively depleted the ER Ca^2+^ content with the reversible SERCA inhibitor CPA (cyclopiazonic acid, 20 μM), prior to stimulating the LS8, ALC and HEK-293 cells with mibefradil in a Ca^2+^-free Ringer’s solution (Fig. 3j). CPA triggered a rise in [Ca^2+^]_cyt_ ~ 200–400 nM (Fig. 3j-k) in all cells. After ER-Ca^2+^ depletion, mibefradil (100 μM) perfusion had no effect in rising [Ca^2+^]_cyt_ (Fig. 3l). Similar results were observed when ER-Ca^2+^ depletion was elicited by stimulation with ATP prior to the application of mibefradil in a Ca^2+^-free Ringer’s solution, as shown in the ALC cells (Fig. S4). These results suggest that ER-Ca^2+^ stores are an important contributor to the effects of mibefradil.

### PLC function is modulated by mibefradil

**Fig. 4:**
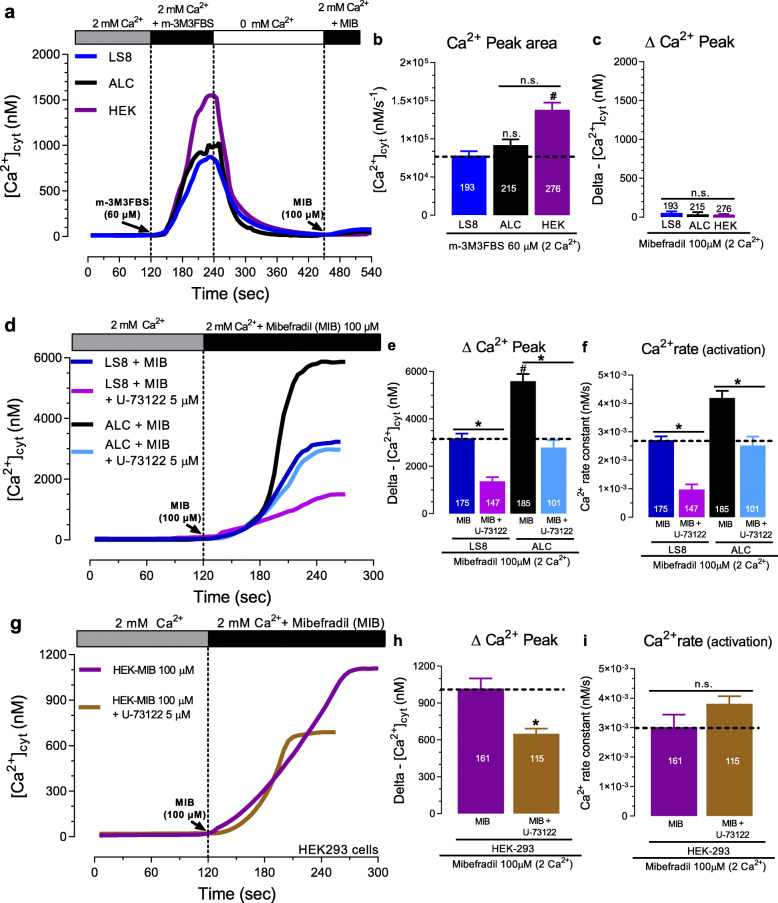
Mibefradil-mediated [Ca^2+^]_cyt_ increase is dependent on PLC pathway activation. **a** Original traces of [Ca^2+^]_cyt_ transients in LS8, ALC, and HEK-293 cells stimulated with the PLC activator m-3M3FBS (60 μM), followed by addition of mibefradil (100 μM) in Ringer’s solution containing 2 mM Ca^2+^. **b** Quantification of the ∆Ca^2+^ peak area of under m-3M3FBS stimulation. **c** Quantification of the ∆Ca^2+^ peak under mibefradil stimulation. **d** Original traces of [Ca^2+^]_cyt_ transients in LS8 and ALC cells stimulated with mibefradil (100 μM) in the presence or absence of the PLC inhibitor, U73122 (5 μM). **d** Quantification of the ∆Ca^2+^ peak. **f** Quantification of the Ca^2+^ influx rate. **g-i** Same as d-f but in HEK-293 cells. Data represent the mean ± SEM of ≥101 cells from 3 independent experiments. Data were analyzed by one-way ANOVA followed by Tukey’s multiple comparison post-hoc test. ^#^*P* < 0.05 vs. LS8 group; *P < 0.05 vs. their respective MIB-100 μM group; n.s., non-significant

We first addressed the gene expression levels of the primary (β-γ) and secondary (δ) PLC isoforms. We found that the mRNA encoding PLC-γ1 was the most highly expressed in LS8 and HEK-293 cells, whereas in ALC cells, the β-γ isoforms were dominant (Fig. S5). Next, to more directly address the mechanism responsible for the [Ca^2+^]_cyt_ elevation by mibefradil, we investigated the functional role of the PLC pathway using the selective PLC activator m-3M3FBS (60 μM) and the PLC inhibitor U73122 (5 μM). In the presence of 2 mM Ca^2+^ solution, m-3M3FBS elicited a significant increase in [Ca^2+^]_cyt_ in all three cell lines, with the highest increase in the Ca^2+^ peak area observed in HEK-293 cells (Fig. 4a-b). After activating PLC with m-3M3FBS, the subsequent perfusion of mibefradil (100 μM) showed only a minimal (≤ 50 nM) effect on [Ca^2+^]_cyt_ (Fig. 4c). Similarly, our data revealed that the PLC blocker U73122 (5 μM, 2 min) reduced the ∆ Ca^2+^ peak in LS8 (~ 65%), ALC (~ 54%), and HEK-293 (~ 35%) cells (Fig. 4d-e, g-h). With the exception of the HEK-293 cells, the PLC blocker also caused a slowdown in the Ca^2+^ influx rate in LS8 and ALC cells (Fig. 4f, i). Taken together, these results support the participation of the PLC pathway and IP_3_Rs in the [Ca^2+^]_cyt_ transients elicited by mibefradil.

### Mibefradil triggered SOCE mediated by ORAI

**Fig. 5:**
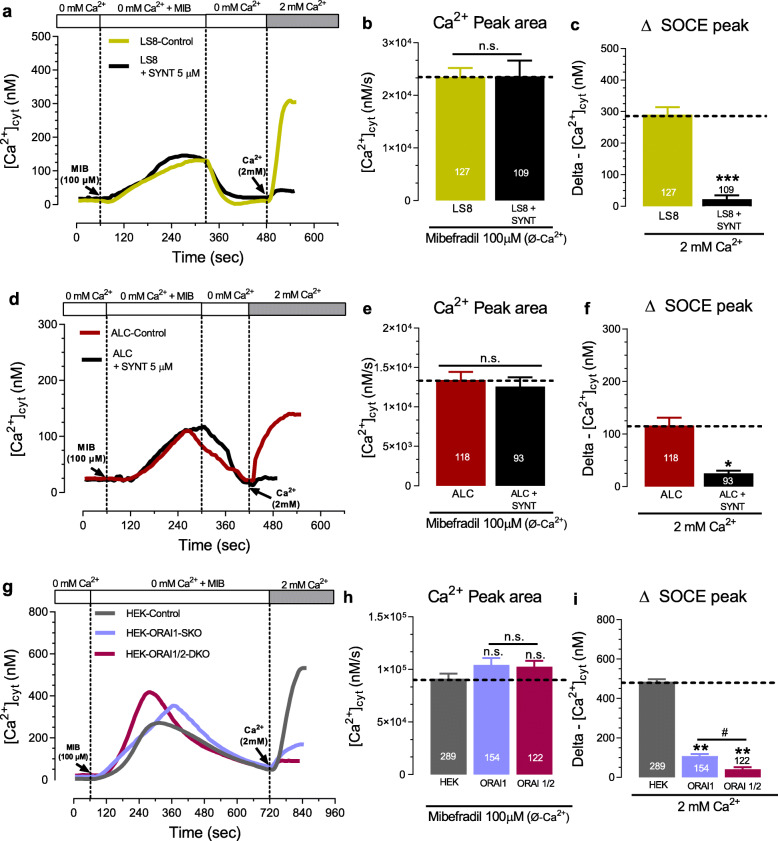
Mibefradil stimulated ER-Ca^2+^ depletion and Ca^2+^ influx via SOCE. **a** Original traces of [Ca^2+^]_cyt_ transients showing ER-Ca^2+^ depletion elicited by mibefradil (100 μM) in Ca^2+^-free Ringer’s solution followed by readdition of 2 mM extracellular Ca^2+^ solution in LS8 cells with or without pre-incubation with synta-66. **b** Quantification of the ER-Ca^2+^ release peak area under mibefradil stimulation. **c** Quantification of ∆SOCE peak upon readdition of 2 mM Ca^2+^. **d** Same as in a-c but in ALC cells. **g** Original traces of [Ca^2+^]_cyt_ transients showing ER-Ca^2+^ depletion elicited by mibefradil (100 μM) in Ca^2+^-free Ringer’s solution followed by readdition of 2 mM extracellular Ca^2+^ solution in HEK-293 cells with a CRISPR-cas9 deletion of *ORAI1* (SKO) or dual deletion of *ORAI2* and *ORAI2* (DKO). **h** Quantification of the ER-Ca^2+^ release peak area under mibefradil stimulation. **i** Quantification of ∆SOCE peak upon readdition of 2 mM Ca^2+^. Data represent the mean ± SEM of ≥93 cells from 3 independent experiments. Data were analyzed by two-tailed unpaired Student’s t-test or one-way ANOVA followed by Tukey’s multiple comparison post-hoc test. *P < 0.05 vs. ALC group; **P < 0.01 vs. HEK-control group; ***P < 0.001 vs. LS8 group; ^#^*P* < 0.05 vs. HEK-ORAI1 group; n.s., non-significant

Because mibefradil was able to release Ca^2+^ from ER stores and this ER-Ca^2+^ efflux is a common step in the activation of SOCE [24], we tested whether treatment with mibefradil triggers Ca^2+^ influx through SOCE. First, to exclude the possibility that plasma membrane leaks or Ca^2+^-sensing receptors (CaSRs) were associated with changes in [Ca^2+^]_cyt_, we monitored [Ca^2+^]_cyt_ transients before and after re-addition of 2 mM extracellular Ca^2+^ in cells with replete ER-Ca^2+^ stores. We showed that this had only a minimal effect in rising [Ca^2+^]_cyt_ (≤ 20 nM) (Fig. S6). By contrast, SOCE activation elicited by the re-addition of 2 mM Ca^2+^ in cells with ER-Ca^2+^ stores previously depleted by mibefradil resulted in a rise in [Ca^2+^]_cyt_ in all cells (~ 150–400 nM) (Fig. 5a-i). Next, we addressed if this SOCE effect was mediated by the ORAI channels. LS8 and ALC cells pretreated with the ORAI blocker synta-66 (5 μM, 2 h), or HEK-293 cells with a CRISPR/Cas9 deletion of *ORAI1* or HEK-293 cells lacking both *ORAI1* and *ORAI2* showed nearly abolished [Ca^2+^]_cyt_ uptake when the cells were perfused with 2 mM Ca^2+^ (Fig. 5c, f, i). These data suggest that mibefradil can elicit the activation of SOCE mediated by ORAI.

## Discussion

Ca^2+^ is a key ubiquitous intracellular messenger coupling membrane-mediated processes to downstream cellular responses [32]. Therefore, Ca^2+^-mediated processes have been studied for decades in clinical and pharmacological settings [33]. Especially relevant is the use of Ca^2+^ channel antagonists (CCA), which dilate the arteries and lower blood pressure, being effective in the treatment of angina or cardiac dysrhythmias [34]. Mibefradil was launched as a new potent Ca^2+^ antagonist in 1997 to inhibit T-type (Ca_v_ 3.1–3.3) channels and was used in the treatment of hypertension and angina [4]. Although mibefradil was a potent and selective Ca_v_ 3.1–3.3 CCA at nanomolar dosage, because of its side-effects and interactions with β-blockers, it was withdrawn from the market in 1998 [35, 36]. However, because T-type (Ca_v_ 3.1–3.3) channels are key regulators of cancer cell proliferation and solid tumor growth [37–39], mibefradil has been recently repurposed as an anticancer drug.

Mibefradil is chemically distinct from other T-type CCA and it is 30-fold more potent against T-type Ca_v_ channels than L-type channels at nanomolar concentrations [4]. The anti-proliferative and anti-tumoral properties of mibefradil are in part associated with lowering AKT phosphorylation and the nuclear retention of FOX [40]. Mibefradil reduced tumor growth in resistant melanoma cells and induced apoptosis via inhibition of autophagy also activating the caspase cascade pathway [16, 41, 42]. These results suggest that the inhibition of autophagy by mibefradil might be a novel therapeutic tool to drive apoptosis and to reduce migration, proliferation, and tumor growth.

Although mibefradil is emerging as an anticancer drug, its pharmacokinetic profile for cancer management is not fully understood. Phase I clinical trials showed the maximum tolerated dose (MTD) by dosing 25–350 mg/day of mibefradil in four doses/day for 7–17 consecutive days [6]. This study reported that the AUC (area under the curve) of mibefradil in plasma for the final dose given on day 8 was 7797 ± 1323 ng h/mL [6], equivalent to ~ 11–16 μM. Mibefradil is efficiently processed by cytochrome P450-catalysed hydrolysis and its metabolites typically represent 50–80% of the circulating drug-related compounds after a single oral dose of 100 mg [5]. Considering that our in vitro studies showed a LogEC_50_ around 45–50 μM for mibefradil and that micromolar (≥ 10 μM) concentrations significantly raised [Ca^2+^]_cyt_ (Fig. 1), understanding the potential cellular targets modulated by mibefradil in vitro affecting [Ca^2+^]_cyt_ should be addressed.

At micromolar concentrations, mibefradil (10 μM) increased [Ca^2+^]_cyt_ in mammalian spermatozoa [18]. Similarly, in HEK-293, rat fibroblasts, and human platelet cells, 10 to 100 μM of mibefradil substantially elevated [Ca^2+^]_cyt_ by unknown mechanisms [19]. Mibefradil was also reported recently to block the Ca^2+^ channel ORAI [25]. Given the limited understanding of the mechanisms by which mibefradil elevates [Ca^2+^]_cyt_, we have investigated a numbers of potential cellular targets and have studied their effects on Ca^2+^ uptake by SOCE mediated by the ORAI channels. We show that the transient perfusion of mibefradil elevates [Ca^2+^]_cyt_ in all cell lines tested (LS8, ALC, and HEK-293) with a concentration-dependent manner and LogEC_50_ around 45–50 μM. Its efficiency to elevate [Ca^2+^]_cyt_ was fully reversed by perfusion of a solution without mibefradil (Fig. 1, Fig. S2).

In a wide variety of cells, the binding of agonists to transmembrane receptors triggers changes in the electrochemical driving force and can depolarize the plasma membrane increasing [Ca^2+^]_cyt_ by activation of multiples second messengers and downstream pathways [34]. One of these transmembrane receptors is the chanzyme TRPM7 that is permeable to divalent cations (Ca^2+^, Mg^2+^, Mn^2+^), and has been linked to the increase of [Ca^2+^]_cyt_ triggered by mibefradil [20, 43]. A previous report showed that mibefradil (100 μM) increased [Ca^2+^]_cyt_ in HEK-293 cells overexpressing TRPM7 channels and that the TRPM7 antagonist NS8593 reduced the effects of mibefradil [20]. Thus, to ascertain if TRPM7 channels are a potential target of mibefradil, we stimulated the cells with the selective TRPM7 agonist naltriben. Although all cells showed the expression of *TRPM7* mRNA, the activation of TRMP7 by naltriben (100 μM, for 5 min) in a Ringer’s solution containing 2 mM Ca^2+^ only elicited a small elevation in [Ca^2+^]_cyt_ (∼30 nM) in all cell lines (Fig. S3). These results suggest that TRPM7 channels are not the main target responsible for the changes in [Ca^2+^]_cyt_ triggered by mibefradil.

Our functional and molecular data revealed that the capacity of mibefradil to elevate [Ca^2+^]_cyt_ is associated with PLC and IP_3_ signaling. We found that the transient application of mibefradil (100 μM for 3–4 min) increased ∼2–6 fold the total IP_3_ content in cells (Fig. 2a-c). The binding of IP_3_ to IP_3_R activates the release of ER-Ca^2+^ pools. At nanomolar (500 nM) concentrations, mibefradil was reported to have no effect on the ER-Ca^2+^, basal Ca^2+^ levels, or on the frequency of Ca^2+^ oscillations [21]. However, Eberhard et al. showed that a substantial part of the mibefradil-induced increase in [Ca^2+^]_cyt_ at micromolar concentrations (10–100 uM) was independent of extracellular Ca^2+^ influx [19], suggesting that a potential source could be from intracellular Ca^2+^ stores. We thus tested if altering intracellular Ca^2+^ by blocking IP_3_R with Xestospongin C modified the effects of mibefradil on [Ca^2+^]_cyt_. In the presence of the IP_3_R inhibitor, the effects of mibefradil were markedly reduced (Fig. 3). We also showed that the prior depletion of ER-Ca^2+^ with CPA completely abolished the effects of mibefradil in [Ca^2+^]_cyt_ (Fig. 3j-l). Combined, these results suggest that the elevations in [Ca^2+^]_cyt_ induced by mibefradil are not only dependent on extracellular Ca^2+^ but also on the ER-Ca^2+^ stores. These data are in line with a previous report in rat fibroblasts and human platelets describing that mibefradil (10 μM) caused the activation of IP_3_R [19]. Our results are also consistent with reports indicating that perfusion of permeabilized cells with IP_3_ prevented a rise in [Ca^2+^]_cyt_ elicited by mibefradil [19].

In non-excitable and excitable cells, the Ca^2+^-dependent PLC pathway is essential to generate second messengers such as IP_3_ and DAG that support the maintenance of [Ca^2+^]_cyt_ homeostasis [22, 23]. We showed that the main PLC isozymes (β, γ, and δ) were expressed in the three cell lines sampled (Fig. S5). We also demonstrated that, under physiological extracellular Ca^2+^ levels (2 mM Ca^2+^), the prior activation of PLC with m-3M3FBS or inhibition of PLC with U73122, significantly reduced the effects of mibefradil (Fig. 4). Taken together, our data strongly support the role of PLC as an important mediator of the effects of mibefradil in elevating [Ca^2+^]_cyt_.

The depletion of ER-Ca^2+^ stores elicited by micromolar concentration (100 uM) of mibefradil raised the possibility that it could also trigger Ca^2+^ influx by SOCE through ORAI channels. Our results confirmed that the increase in Ca^2+^ influx in all cell lines was mediated by SOCE because pretreatment with the ORAI channel blocker synta-66 abolished Ca^2+^ uptake. Moreover, CRISPR/Cas9 knockdown of *ORAI1* or double deletion of *ORAI1/ORAI2* in HEK-293 cells showed that mibefradil could not elevate [Ca^2+^]_cyt_ (Fig. 5g-i). These pharmacological and molecular approaches reveal that ER-Ca^2+^ depletion elicited by mibefradil causes an increase of the [Ca^2+^]_cyt_ by Ca^2+^ influx via SOCE mediated by the ORAI channels. We suggest that this is an indirect effect on ORAI channels because mibefradil directly or indirectly activates PLC and/or IP_3_ signaling.

Our data may be at odds with a previous report that mibefradil blocked the ORAI channels [25]. In overexpressing STIM1/ORAI1–3 HEK-293 cells, mibefradil (10–100 μM) blocked ORAI currents in whole-cell patch-clamp, and 24 h pretreatment with mibefradil (100 μM) abolished ER-Ca^2+^ release and SOCE [25]. By contrast, the same pretreatment using 0.1 to 50 μM of mibefradil did not interfere in the ER-Ca^2+^ or STIM1 translocation [25]. Also, 20 μM mibefradil increased [Ca^2+^]_cyt_ mediated by SOCE [26]. Reports using PHM1–41 cells showed that the acute perfusion of mibefradil at 1 μM did not change the peak of SOCE [44]. Our approach differed in that we investigated the effects of acute mibefradil perfusion, which activated SOCE. Only cells lacking functional ORAI channels could not be positively stimulated by mibefradil.

In summary, we found that mibefradil elicited a concentration-dependent increase in [Ca^2+^]_cyt_. This increase was associated with ER-Ca^2+^ release through IP_3_R channels, PLC pathway activation, and triggered Ca^2+^ influx by SOCE mediated by ORAI channels. Our findings add important knowledge on the effects of mibefradil that may be of basic and clinical relevance as we provide novel targets and insights of mibefradil which should be considered in it is renew clinical use.

## Materials and methods

### Cell culture and genetic knockout of ORAI

The murine epithelial LS8 and ALC cell lines [45] were maintained in Dulbecco’s modified Eagle’s medium (DMEM) containing 10% fetal bovine serum (FBS) and 1% Penicillin-Streptomycin. All reagents were purchased from Thermo Fisher (USA). We also used HEK-293 cells, including single (*ORAI1*) and double (*ORAI1/ORAI2*) knockdowns by CRISPR/Cas9, originally reported in [46], a kind gift by Dr. Mohamed Trebak. All cells were maintained in a humidified CO_2_ incubator under standard conditions (at 37 °C in a 5%-CO_2_), plated on 25 mm borosilicate coverslips (Fisher Scientific, USA) coated with 0.01% Poly-L-lysine (Sigma-Aldrich, USA), and used within 24 h after culture.

### [Ca^2+^]_cyt_ measurements

[Ca^2+^]_cyt_ measurements were performed as previously described [28]. Briefly, cells were incubated for 1 h at room temperature with 1 μM of the ratiometric Ca^2+^ probe Fura-2-AM (Thermo Fisher, USA) in normal Ringer’s solution (pH 7.4) as follows (mM): 155.0 NaCl; 4.5 KCl; 2.0 CaCl_2_; 1.0 MgCl_2_; 10 D-glucose; and 10 HEPES (Sigma-Aldrich, USA). Fluorescence recordings were obtained by 1) a (Nikon Ti2-E Eclipse, Japan) inverted light microscope equipped with an objective (Nikon S Fluor × 20; numerical aperture: 0.75) 2) and a digital SLR camera (DS-Qi2; Nikon, Japan) controlled by computer software (NIS Elements version 5.20.01, USA). Cells were continuously perfused by a six- or eight-way perfusion system (VC-6/8 valve controller) at 5–6 ml per minute with a common outlet 0.28-mm tube driven by electrically controlled valves (Harvard Bioscience Inc., USA). A normal Ringer’s solution or a Ca^2+^-free solution (the same normal Ringer’s solution, however, without 2 mM of Ca^2+^) was used to dissolve all drugs at room temperature. Fura-2-AM was excited alternatively at 340 and 380 nm using a Lambda LS xenon-arc lamp (Sutter Instrument, USA) or pE-340 fura (Cool Led, USA). Emitted fluorescence was collected through a 510 nm emission filter. Fluorescence images were generated at 5-s intervals and the ratio values were calculated. A Fura-2 calcium imaging calibration kit (Thermo Fisher, USA) was used to estimate the [Ca^2+^]_cyt_, according to the manufacturer’s specifications, as previously described [28]. Standard control buffer (background fluorescence), zero free-Ca^2+^ buffer (free-Ca^2+^), and 39 μM free-Ca^2+^ buffer (saturating Ca^2+^) were used to convert the emission ratio at 340/380 nm excitation to estimate the free [Ca^2+^]_cyt_.

### Pharmacology and extracellular Ca^2+^ conditions

To construct mibefradil concentration-effect curves, cells were continuously perfused for 120 s in normal Ringer’s solution (with regular extracellular Ca^2+^ levels, 2 mM Ca^2+^) followed by the addition of 0.1 μM to 100 μM of mibefradil (Tocris Bioscience, USA) for 120 s. TRPM7 channel function was analyzed by perfusing the cells with the selective TRPM7-gating modulator naltriben (100 μM, Tocris Bioscience, USA) in a regular extracellular Ca^2+^ solution, as reported [28]. The effects of mibefradil on [Ca^2+^]_cyt_, were determined in normal or Ca^2+^-free Ringer’s solution supplemented with EGTA (3 mM, Sigma-Aldrich, USA). The role of IP_3_R was analyzed by pretreatment with the selective IP_3_R inhibitor Xestospongin C (3 μM, Tocris Bioscience, USA) for 20 min [30] prior to the application of mibefradil (100 μM). To deplete the ER-Ca^2+^ stores, cells were perfused with the reversible Ca^2+^-ATPase (SERCA) inhibitor cyclopiazonic acid-CPA (20 μM, Sigma-Aldrich, USA), followed by the addition of mibefradil 100 μM in Ca^2+^-free Ringer’s solution. To address the role of the PLC isoenzymes, cells were perfused with the PLC activator m-3M3FBS (60 μM) (Sigma-Aldrich, USA) as reported [47] followed by the addition of mibefradil 100 μM in regular extracellular Ca^2+^ levels (2 mM Ca^2+^). We also use the PLC blocker U73122 (5 μM) (Sigma-Aldrich, USA) simultaneously with the application of mibefradil (100 μM) [48]. To test if mibefradil stimulates SOCE, cells were continuously perfused with mibefradil (100 μM) in Ca^2+^-free Ringer’s solution followed by a re-addition of 2 mM of extracellular Ca^2+^ in normal Ringer’s solution using the CRAC blocker synta-66 (5 μM, Sigma-Aldrich, USA) for 2 h, as previously reported [28] or in HEK-293 cells with a CRISPR/Cas9 deletion of *ORAI1* or a combined *ORAI1* and *ORAI2* deletion. All drugs and chemicals were diluted and stored following the manufacturer’s instructions.

### Quantification of IP_3_ content

IP_3_ content was quantitated before and after incubation with mibefradil (100 μM, 3–4 min) using the colorimetric ELISA kit (E4792–100, BioVision incorp., USA) following the manufacturer’s instructions. Briefly, 1.10^6^ cells/mL were lysed and the supernatant was collected to carry out the optical density (OD) ELISA reaction assay. The IP_3_ kit sensitivity is around 9 pg/mL and the concentration of IP_3_ in the lysed cells can be calculated by comparing the OD of the samples to the standard curve (Fig. S7).

### Real-time PCR

Total RNA was isolated using the RNeasy Micro Kit (Qiagen®), as indicated by the manufacturer, followed by reverse transcription using the iScript™ cDNA Synthesis Kit (Bio-Rad, USA). For qRT-PCR, we used the SsoAdvanced™ Universal SYBR® Green Supermix (Bio-Rad, USA) and performed the experiments in a CFX Connect thermocycler (Bio-Rad, USA) *Gapdh* was used as the housekeeping gene. Relative quantification of the gene responsible for encoding IP_3_R subtype 1–3 (*ITPR1–3*) PLC β, γ, and δ (*PLCB1*, *PLCG1*, and *PLCD1*) and TRPM7 (*TRPM7*) were determined by the 2^–(ΔCT)^ method [28]. All primers were used at 0.25 nM, and the forward/reverse sequences and amplicon size are shown in Table S1 (mouse) and Table S2 (human).

### Data analyses and statistics

All data, mathematical analyses, and graphs were analyzed and/or generated using the GraphPad Prism software version 9.0 (Inc., California, USA), as previously described [28]. The ∆ Ca^2+^ peak and ∆ SOCE peak were calculated by subtracting the maximum Ca^2+^ values from basal Ca^2+^ levels before and after pharmacological manipulation. Ca^2+^ peak area was calculated by integrating the [Ca^2+^]_c_ transients versus time under the stimulus duration using Origin Pro 8 software version 8.08 (Northampton, USA). The kinetics of the rate of Ca^2+^ activation were calculated in each trace and fitted by the GraphPad Prism software using the one-phase association eq. (Y = IF(X < X0, Y0, Y0 + (Plateau-Y0)*(1 - exp.(−K*(X-X0)))). Concentration-effect (0.1 μM to 100 μM) curves for mibefradil were constructed using sigmoidal log (agonist) vs. response - variable slope equation fitted by the GraphPad Prism software and parameter of the LogEC_50_ (apparent affinity) was calculated. Data represent the mean ± SEM of the minimum of three independent experiments. The total number of cells used is indicated. Differences between the means of the group data that fit a normal distribution were analyzed using a two-tailed unpaired Student’s t-test variance or by one-way ANOVA followed by Tukey’s multiple comparisons post-hoc test. The limit of significance was established at *p < 0.05; **p < 0.01; ***p < 0.001.

## Supplementary Information

**Additional file 1: Figure S1.** Effects of Ringer’s solution and mibefradil (50–100 μM) on background autofluorescence. **Figure S2.** Effects of the two consecutive mibefradil (50 μM) stimulus on [Ca^2+^]cyt transients in HEK-293 cells under regular extracellular Ca^2+^ levels (2 mM Ca^2+^). **Figure S3.** TRMP7 expression and activation by naltriben. **Figure S4.** Alternative stimulation with mibefradil (100 μM) and ATP (100 μM) under Ca^2 +^ −free Ringer’s solution in ALC cells. **Figure S5.** Expression of PLC isoforms. **Figure S6.** Effects of the Ca^2 +^ −free Ringer’s solution replacement for 2 mM extracellular Ca^2+^ in LS8, ALC, and HEK-293 cells. **Figure S7.** Optical density (OD) values of standard IP3 concentration. **Table S1.** Mouse primers sequences for qRT-PCR used in LS8 and ALC cells. **Table S2.** Human primers sequences for qRT-PCR used in HEK-293 cells.

## Data Availability

The data is available with the corresponding author and will be provided upon a legitimate request.
