# Supplementary material for: Mibefradil alters intracellular calcium concentration by activation of phospholipase C and IP3 receptor function
Source: Mol Biomed. 2021 Apr 30;2:12. doi: 10.1186/s43556-021-00037-0 (PMC8607413; doi:10.1186/s43556-021-00037-0)
Supplement: Supplementary file 1 — Additional file 1: Figure S1. Effects of Ringer’s solution and mibefradil (50–100 μM) on background autofluorescence. Figure S2. Effects of the two consecutive mibefradil (50 μM) stimulus on [Ca2+]cyt transients in HEK-293 cells under regular extracellular Ca2+ levels (2 mM Ca2+). Figure S3. TRMP7 expression and activation by naltriben. Figure S4. Alternative stimulation with mibefradil (100 μM) and ATP (100 μM) under Ca2 + −free Ringer’s solution in ALC cells. Figure S5. Expression of PLC isoforms. Figure S6. Effects of the Ca2 + −free Ringer’s solution replacement for 2 mM extracellular Ca2+ in LS8, ALC, and HEK-293 cells. Figure S7. Optical density (OD) values of standard IP3 concentration. Table S1. Mouse primers sequences for qRT-PCR used in LS8 and ALC cells. Table S2. Human primers sequences for qRT-PCR used in HEK-293 cells. [file 43556_2021_37_MOESM1_ESM.pdf]

Supplementary Materials for

Mibefradil alters intracellular calcium concentration by activation of phospholipase C and IP_3 receptor function

Guilherme H. Souza Bomfim[a], Erna Mitaishvili[a], Talita Ferreira Aguiar[b], Rodrigo S. Lacruz[a*]

a. Department of Molecular Pathobiology, New York University College of Dentistry, New York, NY 10010, USA.
b. Department of Urology, New York University School of Medicine, New York, NY 10010, USA.

***** Corresponding author: Rodrigo S. Lacruz; Department of Basic Science and Craniofacial Biology, New York University College of Dentistry, New York, NY 10010, USA.

E-mail addresses: rodrigo.lacruz@nyu.edu; rsl10@nyu.edu

Includes:

Fig. S1. Effects of Ringer's solution and mibefradil (50-100 µM) on background autofluorescence.

Fig. S2. Effects of the two consecutive mibefradil (50 µM) stimulus on $[Ca^{2+}]_{cyt}$ transients in HEK-293 cells under regular extracellular Ca^{2+} levels (2 mM Ca^{2+}).

Fig. S3. *TRMP7* expression and activation by naltriben.

Fig. S4. Alternative stimulation with mibefradil (100 µM) and ATP (100 µM) under Ca^{2+}-free Ringer's solution in ALC cells.

Fig. S5. Expression of PLC isoforms.

Fig. S6. Effects of the Ca^{2+}-free Ringer's solution replacement for 2 mM extracellular Ca^{2+} in LS8, ALC, and HEK-293 cells.

Fig. S7. Optical density (OD) values of standard IP3 concentration.

Table S1. Mouse primers sequences for qRT-PCR used in LS8 and ALC cells.

Table S2. Human primers sequences for qRT-PCR used in HEK-293 cells.

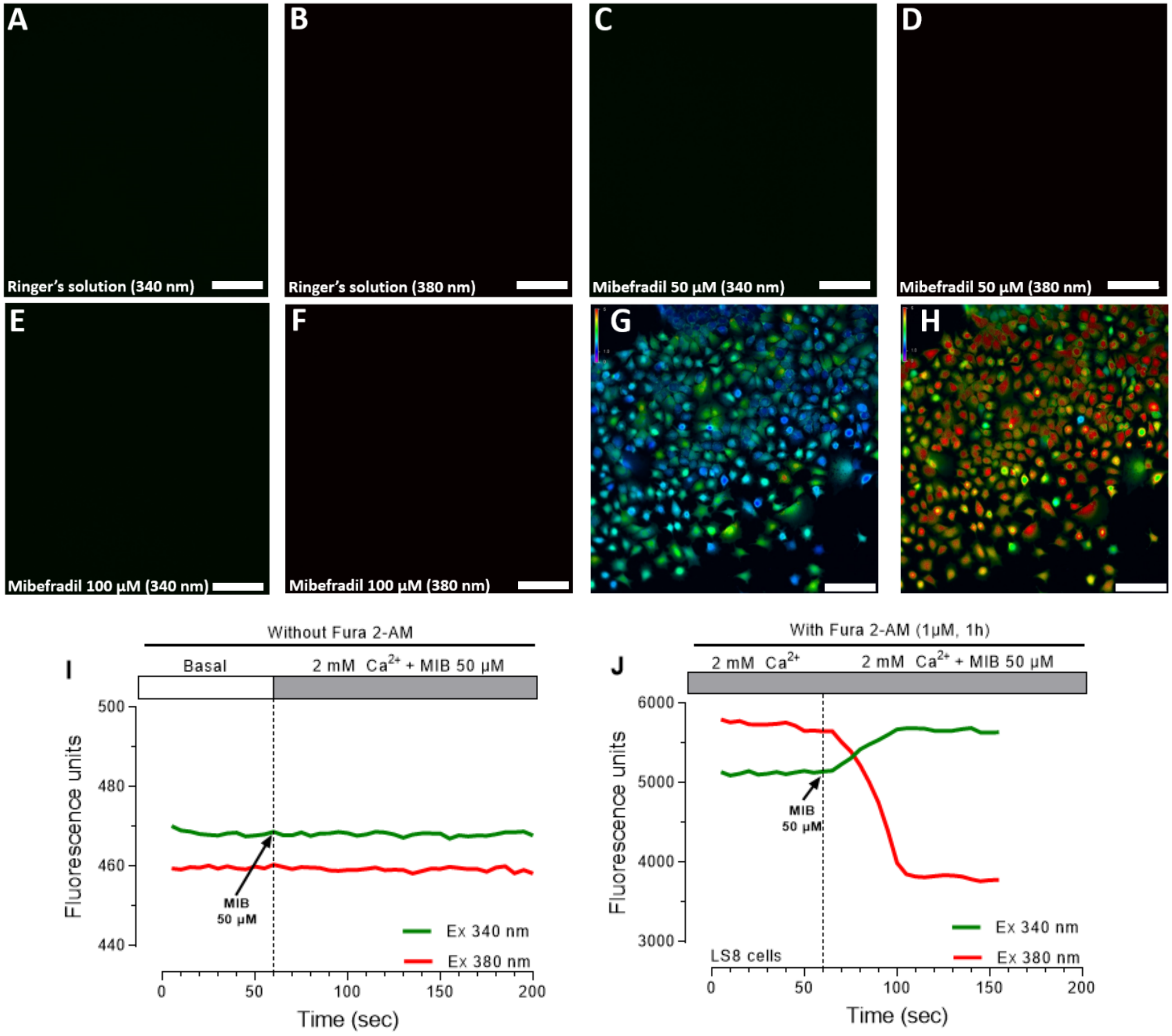

Fig. S1. Effects of Ringer's solution and mibefradil (50-100 μM) on background autofluorescence.

The following solutions were analyzed for autofluorescence by exciting alternatively at 340 and 380 nm: **(A-B)** Standard Ringer's solutions (2 mM Ca^{2+}), **(C-D)** Ringer's solutions containing mibefradil (50 μM) **(E-F)** Ringer's solutions containing mibefradil (100 μM). **(G-H)** Representative images of $[Ca^{2+}]_{cyt}$ transients in ALC cells before **(G)** after **(H)** perfusion with mibefradil (100 μM) in normal Ringer's solution (2 mM Ca^{2+}). **(I)** Original traces of LS8 cells excited alternatively at 340 (green) nm and 380 (red) nm before and after the addition of mibefradil (50 μM) in a Ringer's solution without Fura 2-AM or loaded with Fura 2-AM **(J)**. Scale bar: 100 μm. Nikon S Fluor × 20; numerical aperture: 0.75.

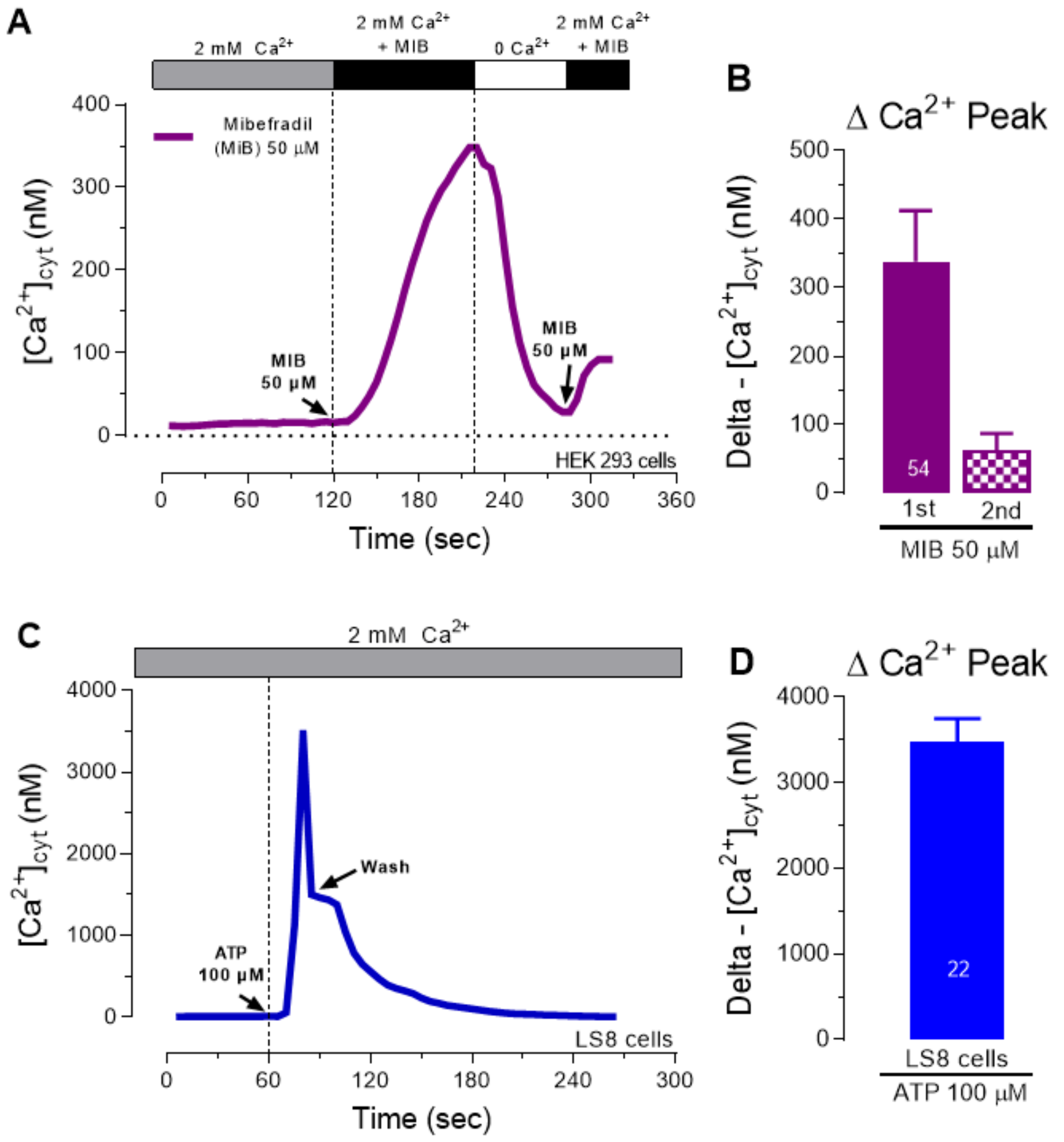

Fig. S2. Effects of two consecutive mibefradil (50 μM) or ATP (100 μM) stimulus on $[Ca^{2+}]_{cyt}$ transients under regular extracellular Ca^{2+} levels (2 mM Ca^{2+}).

(A) Original traces of $[Ca^{2+}]_{cyt}$ transients after two consecutive mibefradil (50 μM) stimulations in regular extracellular Ca^{2+} levels (2 mM Ca^{2+}). **(C)** Original traces of $[Ca^{2+}]_{cyt}$ transients after a single ATP (100 μM) stimulus in regular extracellular Ca^{2+} levels (2 mM Ca^{2+}). **(B)** ΔCa^{2+} peak for two mibefradil stimulus in HEK-293 cells. **(D)** ΔCa^{2+} peak for ATP stimulus in LS8 cells. Data represent the mean ± SEM of ≥ 22 cells from 2 independent experiments. The number of cells used in each experiment is shown in the histograms.

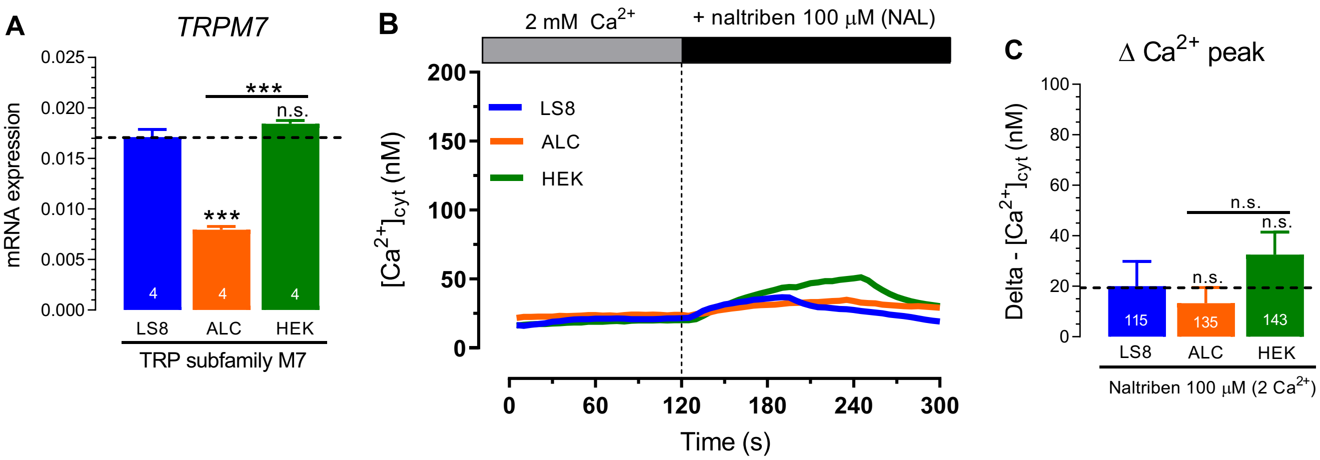

Fig. S3. *TRMP7* expression and activation by naltriben.

(A) RT-PCR analyses showing the expression of *TRPM7* mRNA in LS8, ALC, and HEK-293 cells. **(B)** Original traces of $[Ca^{2+}]_{cyt}$ transients after perfusion with the selective TRPM7 agonist naltriben (100 µM) in regular extracellular Ca^{2+} levels (2 mM Ca^{2+}) in LS8, ALC, and HEK-293 cells. **(C)** ΔCa^{2+} peak for each cell type. Data represent the mean ± SEM of ≥ 115 cells from 3-4 independent experiments analyzed by one-way ANOVA followed by Tukey's multiple comparisons post-hoc test. n.s., non-significant. Number of cells used in each experiment is shown in the histograms. ***P < 0.001 vs. LS8 or ALC groups; n.s., non-significant.

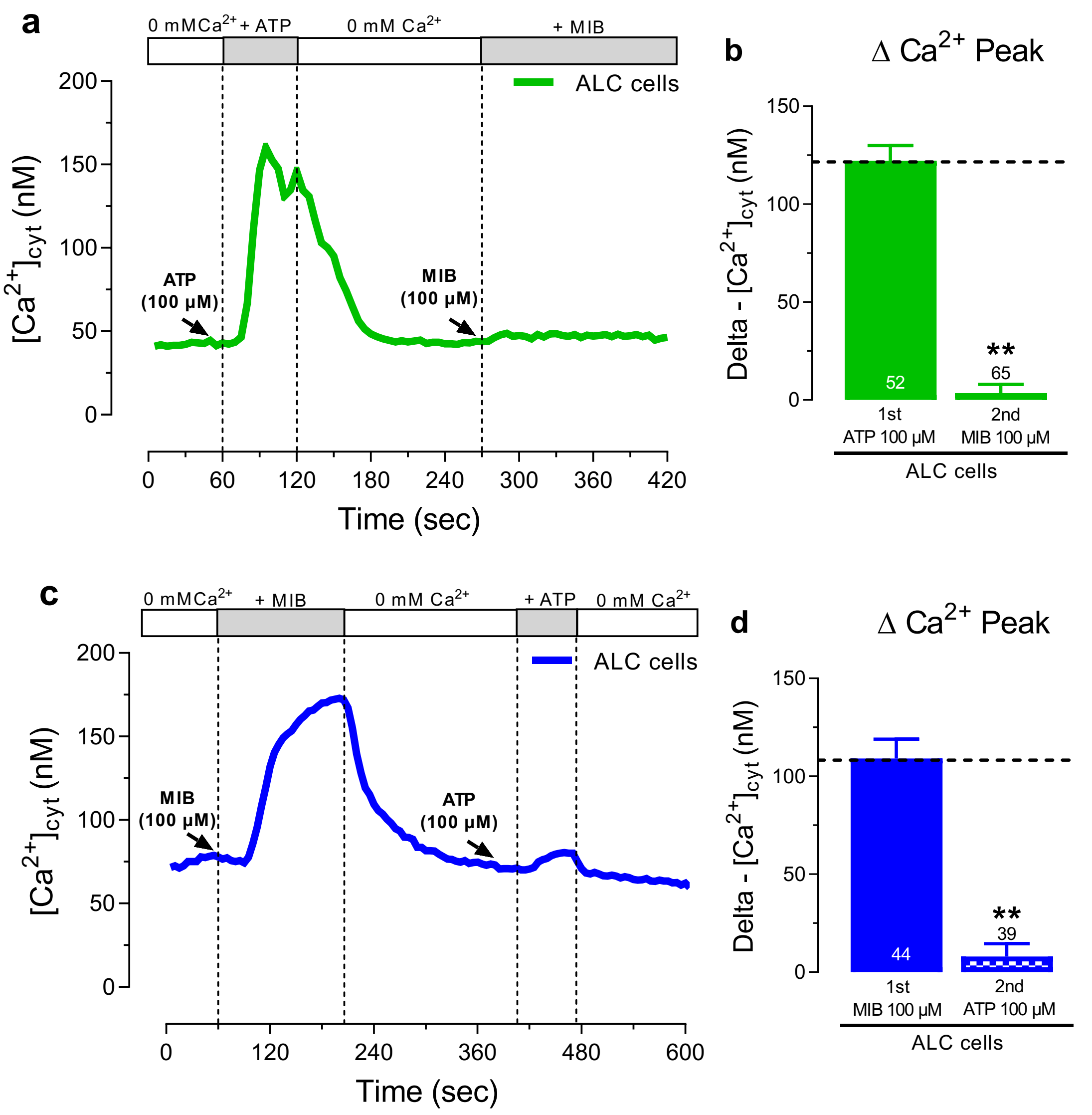

Fig. S4. Alternative stimulation with mibefradil (100 μM) and ATP (100 μM) under Ca^{2+}-free Ringer's solution in ALC cells.

(A) Original traces of $[Ca^{2+}]_{cyt}$ transients after mibefradil (100 μM) stimulation followed by and ATP (100 μM) (B) Quantification of ΔCa^{2+} peak. (B) Original traces of $[Ca^{2+}]_{cyt}$ transients after ATP (100 μM) stimulation followed by mibefradil (100 μM) **(D)** Quantification of ΔCa^{2+} peak. Data represent the mean ± SEM of ≥ 39 cells from 2 independent experiments. The number of cells used in each experiment is shown in the histograms. Data were analyzed by two-tailed unpaired Student's t-test. $^{**}P < 0.01$ vs. respective 1st stimulus (control) group.

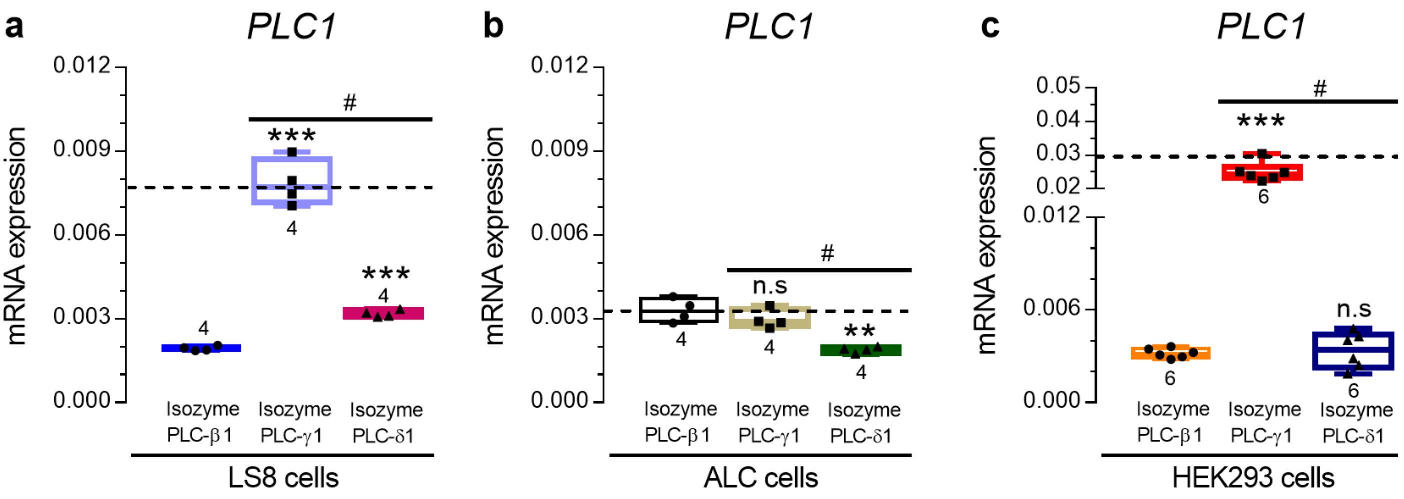

Fig. S5. Expression of PLC isoforms

(A-C) Relative mRNA expression of the gene encoding PLC isoforms *PLC-β1 PLC-γ1* and *PLC-δ1* quantified by qPCR in LS8, ALC, and HEK-293 cells. Data represent the mean ± SEM of 4-6 independent experiments. Data were analyzed by one-way ANOVA followed by Tukey's multiple comparison post-hoc test. $^{**}P < 0.01$ or $^{***}P < 0.001$ vs. PLC-β1 group; $^{\#}p < 0.05$ vs. PLC-γ1 group; n.s., non-significant.

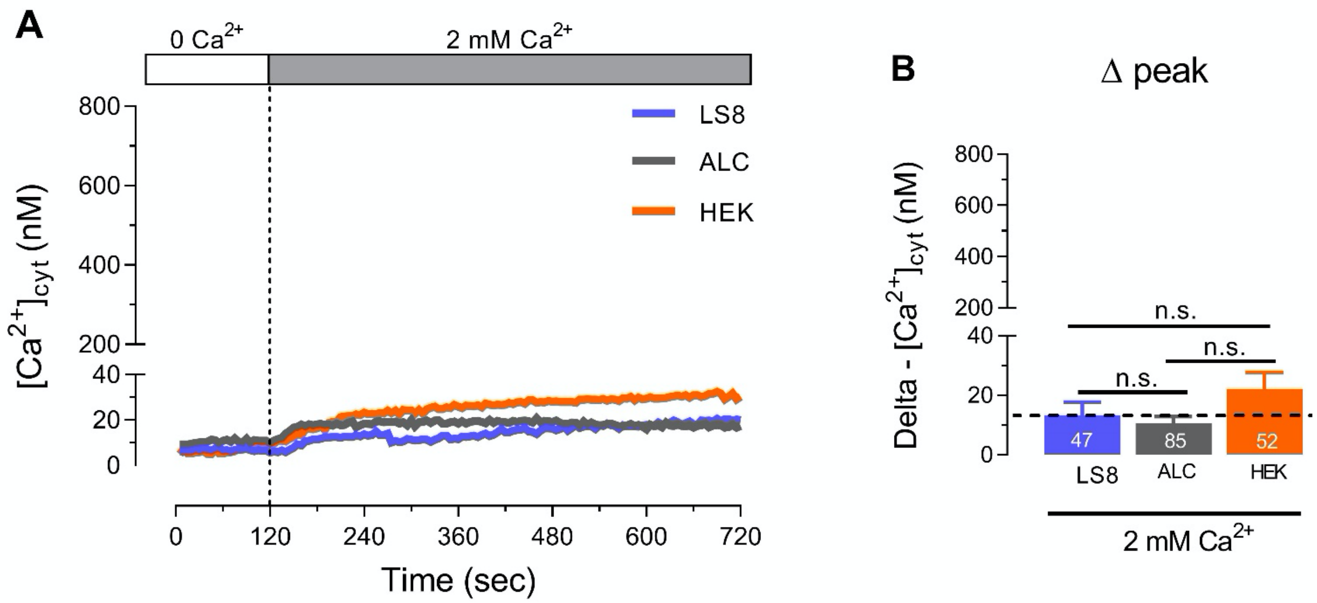

Fig. S6. Replacing Ca^{2+}-free Ringer's solution with 2 mM had no effect on $[Ca^{2+}]_{cyt}$

(A) Original traces of $[Ca^{2+}]_{cyt}$ transients before and after re-addition of 2 mM extracellular Ca^{2+} to measure the Ca^{2+} uptake potentially mediated by plasma membrane leak and Ca^{2+}-sensing receptors (CaSR) in LS8, ALC, and HEK-293 cells. **(B)** Δ Ca^{2+} peak for each cell type. Data represent the mean ± SEM of ≥ 47 cells from 2-3 independent experiments analyzed by one-way ANOVA followed by Tukey's multiple comparisons post-hoc test. n.s., non-significant. Number of cells used in each experiment is shown in the histograms.

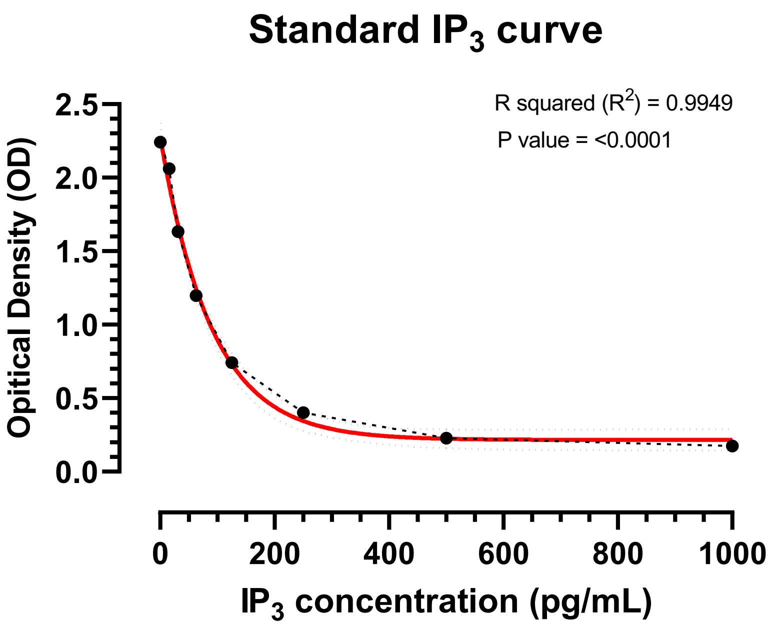

Fig. S7. Optical density (OD) values of standard IP_3 concentration

Average values of duplicate readings for each standard concentration are plotted in the four-parameter logistic curve graph with a concentration on the x-axis and OD values on the y-axis. There is a negative correlation between OD and IP_3 concentration. R squared (R2) values were 0.9949 and P-value <0.0001.

Table S1. Mouse primer sequences for qRT-PCR used in LS8 and ALC cells.

Table 1. List of MOUSE primer couples for IP_3R, PLC and TRPM7.

Gene	Forward sequence (5' → 3')	Reverse sequence (3' → 5')	Amplicon size
Gapdh	GACTTCAACAGCAACTCCCAC	TCCACCACCCTGTTGCTGTA	125
ITPR1	ATCACCATCATGCAGCCTAT	TCTCCAGGGTTTGGTTGATA	229
ITPR2	TGACAGTGATGGACACCAAG	AACATTGTTTCTGCCTGAGC	223
ITPR3	GCGGGAGCAGAACATACTTA	CCAGGATGTCATAGCCAATC	245
PLC β1	CAGAAGTAGAGGCGCAAACC	CTCCGTGGTTTTCTTGTGGT	122
PLC γ1	AGGCAAGAAGTTCCTCCAGT	CTTCGTCTCTCATGGTGCTT	230
PLC δ1	GCCGTCAGACAGCAGTTATT	TACTCTGGCCGATAAAGTCG	181
TRPM7	ATGGCACTGTTGGAAAGTATGG	CGCCTTCAAATATCAAAGCCAC	129

Table S2. Human primers sequences for qRT-PCR used in HEK-293 cells.

Table 2. List of HUMAN primer couples for IP3R, PLC and TRPM7.

Gene	Forward sequence (5' → 3')	Reverse sequence (3' → 5')	Amplicon size
Gapdh	GACAGTCAGCCGCATCTTC	GCGCCCAATACGACCAAAT	104
ITPR1	CCTGGTTGATGATCGTTGTGTT	GCTTTTGGGCAGAGTAGCGGTT	119
ITPR2	AAAGCCTCAGTGGAATCCTGT	ATGGCAATTCCACGATTTTT	65
ITPR3	GGAGCAAGATCGTCCATCA	CTGCTGTAGCCAGTGCAGAC	95
PLC β1	GCTGGAAACTCCTCTGTTGA	CCGCAATTGCTTCTATCACT	169
PLC γ1	GGAGCTCTCTGAACTTGTCG	AGCTGCAGTCGATTGTACTG	171
PLC δ1	AGATCCTCTTCTGCGATGTG	TCTCCAGGGATAGGATGACA	84
TRPM7	GACGTGGTTGACTCCATTTC	GAGGCAGAACTGGTTTCTCA	182
